# Foresight for ethical AI

**DOI:** 10.3389/frai.2023.1143907

**Published:** 2023-07-20

**Authors:** Stephen L. Dorton, Lauren M. Ministero, Balca Alaybek, Douglas J. Bryant

**Affiliations:** ^1^Social & Behavioral Sciences Department, The MITRE Corporation, Bedford, MA, United States; ^2^School of Public Policy, University of Maryland, College Park, MD, United States

**Keywords:** artificial intelligence, naturalistic decision making, ethics, foresight, premortem, policy

## Abstract

There is growing expectation that artificial intelligence (AI) developers foresee and mitigate harms that might result from their creations; however, this is exceptionally difficult given the prevalence of emergent behaviors that occur when integrating AI into complex sociotechnical systems. We argue that Naturalistic Decision Making (NDM) principles, models, and tools are well-suited to tackling this challenge. Already applied in high-consequence domains, NDM tools such as the premortem, and others, have been shown to uncover a *reasonable* set of risks of underlying factors that would lead to ethical harms. Such NDM tools have already been used to develop AI that is more trustworthy and resilient, and can help avoid unintended consequences of AI built with noble intentions. We present predictive policing algorithms as a use case, highlighting various factors that led to ethical harms and how NDM tools could help foresee and mitigate such harms.

## 1. Introduction: ethical AI, policy, and the need for crystal balls

Few topics have received as much attention in the last several years as the development of ethical artificial intelligence (AI). There has been an overwhelming development of frameworks and principles-based guidance toward developing ethical AI, although different frameworks use different terms such as ethical, assured, or trustworthy interchangeably (Blasch et al., 2021; Munn, 2022). Such guidance has been developed by government agencies, non-profits, and private companies with the goal of reducing or eliminating ethical harms associated with development and adoption of AI technologies (Hallamaa and Kolliokoski, 2022).

Despite the proliferation of frameworks and guidelines, they can be difficult to put into practice (Munn, 2022). One outcome of this lack of pragmatism in AI ethics is a phenomenon called ethical debt (Heimo and Holvitie, 2020; Petrozzino, 2021). Ethical debt is analogous to technical debt, where AI tools are developed under the presumption that the AI solution itself is ethical. For the sake of development efficiency and lack of concrete means to address ethical consequences, such consequences often go ignored or unmitigated until ethical harms become manifest. A key aspect of ethical AI that we focus on for the remainder of this manuscript is the responsibility of AI developers to foresee and mitigate harms. This concept is epitomized by the Ad Hoc Committee on Responsible Computing's first rule of responsible computing (Miller, 2011, p. 58):

“The people who design, develop, or deploy a computing artifact are morally responsible for that artifact, and for the foreseeable effects of that artifact. This responsibility is shared with other people who design, develop, deploy or knowingly use the artifact as part of a sociotechnical system.”

This need to foresee and mitigate harms is being codified in part via new legislation, mostly in Europe, that makes it easier for individuals to sue AI developers for harms (Hacker, 2022) or requires AI-driven social platforms to manage harms or face various fines and penalties (Husovec and Laguna, 2022). The United States has echoed these sentiments in the new “AI Bill of Rights,” although there are already criticisms of this policy's limitations (Hine and Floridi, 2023).

Despite the legal incentives, foreseeing and mitigating AI-driven harms is easier said than done given the prevalence of emergent behaviors when AI is integrated into sociotechnical systems. Emergent behaviors or properties can be defined as those arising from interactions among components in a sociotechnical system, that are otherwise not observable when investigating any one component (Carayon et al., 2015). Such behaviors exist at the individual level, where introducing AI into a workflow can increase human complacency (Ezer et al., 2019), or drive workflow adaptations based on changes in trust with the AI over time (Dorton et al., 2022). There is also the issue of complexity at the higher, sociotechnical system level. We have long known that sociotechnical systems will stretch when novel technology is introduced, altering the types of work performed (Woods, 2006; Sheridan, 2008). For example, introducing AI into the domain of intelligence analysis raises questions about the nature of analysts' work changing to focus on inspecting AI outputs vs. conducting analysis, as well as other changes to the nature of workforce collaboration and management (Vogel et al., 2021).

In the AI ethics community, Mittelstadt et al. (2016) have discussed examples of transformative effects where introduction of AI drives humans to re-envision their world, classifying more nuanced phenomena discretely. AI ethicists have acknowledged the need for taking a more holistic view (e.g., Asaro, 2019), arguing for a need to frame ethical discourse on human-AI teams rather than individual agents in simple “trolley problems” that neglect emergent behaviors in complex systems (Borenstein et al., 2019; Cañas, 2022).

These difficulties are echoed in the legal community, where there are concerns about the adequacy of tort, liability, and negligence law to cope with novel AI technology (e.g., see Sullivan and Schweikart, 2019; Selbst, 2020; Stein, 2022). A key issue is that these legal doctrines rely on a *reasonable expectation* of an entity to foresee harms in order to be held liable for them. That is, if one can argue that an AI developer could not reasonably have been expected to foresee an emergent harm, then there is no legal recourse for those harmed by the AI—effectively nullifying new policies to hold AI developers accountable. Hacker et al. (2020) argue that explainability will be required to overcome such issues. We believe explainability alone is insufficient as it does not address the underlying issue of emergence in sociotechnical systems. Along these lines, Selbst (2020) asserts that individuals cannot reasonably be expected to have the capacity to understand AI, regardless of how explainable or interpretable it is. More directly, Cofone (2018) proposes characterizing AI systems by their level of emergence, or degree to which there is predictability of the AI interacting with the environment.

In summary, new regulatory policies increasing liability for AI developers have merit in theory but may be difficult to exercise in practice. They will provide incentive for AI developers to more carefully consider the ethical harms their technologies might cause, changing the calculus of accruing ethical debt for the better. However, the relevant legal constructs to enforce this feedback loop are based on the concept of what harms one may reasonably expect the developer to foresee. Not only is this difficult in practice because of emergent behaviors associated with AI, but it is reactive, requiring harms to occur before the developer is held accountable. Therefore, a major objective going forward should be to increase foresight of ethical harms created by adding AI into complex sociotechnical systems.

## 2. Increasing understanding and foresight

### 2.1. Understanding: naturalistic decision making

Naturalistic decision making (NDM) is the research tradition that studies decision making processes under realistic conditions (Klein et al., 1993; Klein, 2022). NDM focuses on the sensemaking and decision making processes of experienced people in work environments characterized by complexity, uncertainty, and time pressure. NDM has been contrasted to *microcognitive* judgement and decision paradigms based on giving naïve participants artificial tasks in controlled laboratory environments, which are not representative of the operational settings in which findings would be applied (Klein et al., 1993). NDM research has shown that experienced decision makers do not simply choose from a set of options, but instead recognize cues and patterns, diagnose situations as anomalous, and evaluate their options by mental simulation in highly novel situations. NDM accounts for the adaptive and integrative relationships among sensemaking and decision making processes (e.g., Ward et al., 2018), and has proven useful where systems developed based on laboratory decision paradigms have failed (Nemeth and Klein, 2010).

NDM encourages a *macrocognitive* perspective by applying naturalistic inquiry methods to study and support cognitive functions (e.g., sensemaking, planning, and coordinating) and processes (e.g., detecting problems and maintaining common ground), as well as the development of expertise (Hoffman et al., 2014; Naturalistic Decision Making Association (NDMA), n.d.). Given these various aims, NDM is regularly applied to a variety of complex and high-consequence work domains, such as aviation, healthcare, firefighting, intelligence analysis, and military decision making (see Klein and Wright, 2016). Of particular interest here is that the holistic view of macrocognition enables the study of emergent phenomena in sociotechnical systems, such as feedback loops, equilibrium, and self-organization, etc. (Klein et al., 2003). Employment of this macrocognitive perspective has resulted in the development of several tools and methods for improving decisions.

### 2.2. Foresight: the premortem and other NDM tools

A premortem—the opposite of a postmortem—is a lightweight method that has been applied in various high-consequence domains both inside and outside of the NDM community to proactively determine why a project or plan has hypothetically failed (Klein, 2007, 2022; Veinott et al., 2010; Eckert, 2015). To begin a premortem, team members are given two minutes to brainstorm reasons for failure (i.e., project risks). A facilitator then asks each person to share a reason from their list, alternating between participants until all unique reasons have been recorded (Klein, 2007). There are some methodological variants that include generating mitigations for the identified risks (Veinott et al., 2010; Klein, 2022), or a more formal elicitation process aimed at uncovering potential root causes of failure (Eckert, 2015).

A key benefit of the premortem lies not in the proactive enumeration of risks, but in the *types* of risks that are typically identified. With a diverse group of participants, premortems can uncover creative and novel risks that span multiple areas of expertise. Such risks could not be identified by project managers alone (Klein, 2007; Bettin et al., 2022). Further, premortems identify risks at the intersection of humans and technology, which require an interdisciplinary lens to uncover. Bettin et al. (2022) describes how the non-constrained thinking inherent in premortems results in the identification of risks within sociotechnical systems that are often overlooked as they are outside of immediate control, including risks associated with hypothetical user behavior patterns, and those related to broader longitudinal cultural, affective, or motivational factors. This resonates with other work demonstrating that premortems can identify other risks that may not be immediately obvious to a single leader or analyst, including a broad array of personnel- and policy-based risks (Klein, 2007), risks stemming from programmatics and team interactions (Eckert, 2015), and risks stemming from operational, organizational, technical, and business factors (Gallop et al., 2016). These outcomes position the premortem as a potentially invaluable method for predicting ethical harms (i.e. manifestations of risks) resulting from introducing AI into complex sociotechnical systems.

While the premortem is a sound candidate for the challenge that we present, it should be noted that it is not the only NDM approach that could be employed to identify AI-driven harms. Recent work has shown that analytic wargames involving experienced players in naturalistic settings can be used to test disruptive technologies and uncover emergent behaviors (de Rosa and De Gloria, 2021), and to explore how the work system may stretch with the introduction of new technologies (Dorton et al., 2020). Other approaches have included the use of naturalistic methods such as the critical incident technique to develop evidence-based checklists for AI developers (e.g., Dorton, 2022). Further still, new methods based on naturalistic inquiry such as Systematic Contributors and Adaptation Diagramming (SCAD; Jefferies et al., 2022) and Joint Activity Monitoring (JAM; Morey et al., 2022) have been developed to attempt to identify issues in work systems more proactively.

### 2.3. Predictive policing as a use case

“Predictive policing” typically refers to the application of quantitative techniques to direct police intervention to prevent crimes by predicting one or both of the following: Places and times of crimes, and people who are likely to commit crimes (Degeling and Berendt, 2018; Coeckelbergh, 2020; Miró-Llinares, 2020). Such technologies were developed with noble intentions to reduce crime and increase the objectivity and efficiency of policing. However, there is a growing consensus that AI-driven predictive policing technologies are not only of dubious efficacy but also result in numerous ethical harms such as disproportionate surveillance and targeting of specific socioeconomic or racial groups, and threatening privacy and civil liberties (Degeling and Berendt, 2018; Asaro, 2019; Richardson et al., 2019; Coeckelbergh, 2020; Miró-Llinares, 2020). This example may prompt the questions, “How did this go wrong?” and “Why didn't we see this coming?”

The left column in Table 1 provides a non-exhaustive list of various contributing factors to ethical harms in predictive policing (Degeling and Berendt, 2018; Asaro, 2019; Richardson et al., 2019; Miró-Llinares, 2020; Alikhademi et al., 2021). First, police are not trained data scientists, so data may not be correctly and consistently annotated (e.g., approximate or inaccurate time stamps in their reports make models heavily dependent on temporal relationships less reliable). Second, data have been intentionally manipulated in some instances (e.g., asking people not to press charges) to create the appearance of reduced crime rates. Third, AI was fed data that were systematically biased (e.g., from jurisdictions under consent decrees for discriminatory practices), resulting in crime location predictions that simply reflect biases and the deference of judgement to faulty model predictions. Finally, police were not informed about what to do with AI outputs, resulting in inconsistent practices when patrolling AI-designated “high risk” areas.

**Table 1:** Factors driving ethical harms vs. risks foreseen via NDM tools.

**Factors driving ethical harms**	**Relevant risks foreseen via NDM tools**
Police were not skilled in data entry and processing	Premortems (Bettin et al., 2022) have foreseen risks with assumptions of user skills. NDM research-driven AI checklists (e.g., Dorton, 2022) have also highlighted this issue
Data were intentionally manipulated to suppress crime rates	Premortems have foreseen risks from bad faith actors (e.g., Veinott et al., 2010) and from the influence of leadership on policies and practices (e.g., Klein, 2007)
False veneer of objectivism provided to biased data created a self-fulfilling prediction	Wargames (e.g., Dorton et al., 2020) would identify such risks by nature of gameplay
No guidance on how to operationalize system outcomes	Premortems (Bettin et al., 2022) have foreseen risks of users being unfamiliar with systems and not following protocols. Wargames (e.g., Dorton et al., 2020) would identify such risks by nature of gameplay

The right column of Table 1 shows how just a few NDM tools have been shown to proactively identify such risk factors in comparable systems. Of particular interest is that the premortem technique addresses three of the four enumerated (or analogous) factors in this use case, despite there being a relatively sparse body of extant literature on the premortem from which to draw. Therefore, we argue that premortems can proactively identify ethical harms from, or failures of, AI. It seems more likely, however, that premortems and other NDM tools will help AI developers foresee the *underlying factors* that contribute to ethical harms, as shown in this predictive policing example.

## 3. Discussion

Overcoming ethical debt in AI development is a substantial challenge. More specifically, foreseeing and mitigating ethical harms is exceptionally difficult given the prevalence of emergent behaviors that occur when integrating AI into sociotechnical systems. We have argued that the NDM community (as a personification of its people, principles, and tools) is well-suited to tackling this challenge. However, we do caution that the AI ethics community should not view NDM or macrocognitive approaches as a panacea: that is, adding an NDM practitioner to your team or using an NDM method does not guarantee success in foreseeing and mitigating harms.

Premortems do not guarantee an exhaustive set of risks will be identified (Bettin et al., 2022). Similarly, methods such as analytic games (e.g., de Rosa and De Gloria, 2021) and other checklists based on naturalistic inquiry (e.g., Dorton, 2022) are bounded in scope by not only the scenarios and injects examined, but also the expertise of the participants involved in their application. More generally, there is a long-standing challenge of developing tools for an envisioned world with technological change (e.g., Woods and Dekker, 2010). Even newer tools focused on proactive identification of risks such as SCAD (Jefferies et al., 2022) and JAM (Morey et al., 2022) rely on collecting data from *existing* sociotechnical systems, making them insufficient to truly provide foresight *before* integrating new AI into work systems. Thus, future methodological research is needed to develop and refine methods to enable greater foresight of ethical harms. For example, could a premortem be scoped or targeted to foresee ethical harms vs. more general project risks?

While we have provided an argument for how NDM tools can increase *foresight* (i.e., proactively identifying emergent risks), we have not addressed problem solving (i.e., what to do with identified risks). While anecdotal evidence has shown that the outputs of NDM tools such as the premortem can inform requirements, designs, and test and evaluation activities, additional work is needed to more concretely map the outputs of NDM tools like the premortem into the AI development process.

Acknowledging these limitations, we maintain that the use of NDM principles and tools has obvious value to AI developers. This value extends beyond developing *ethical* AI. NDM approaches have been used to glean insights on developing *trustworthy* AI (e.g., Dorton and Harper, 2022 and Dorton, 2022) and *resilient* AI technologies (e.g., Neville et al., 2022). In summary, we have attempted to craft our argument while providing different messages to two different audiences. To the AI ethics community: While NDM is not a panacea, the NDM community has a considerable head start addressing the aforementioned challenges. To the NDM community: There is an opportunity to refine our models and tools to improve foresight and reduce ethical debt in AI. So, can NDM tools uncover an *exhaustive* set of emergent harms for a given AI technology? No. It would be naive to think so. Can NDM tools uncover a *reasonable* set of emergent harms? We believe so.

## Author contributions

SD: conceptualization, investigation, and writing—original draft. LM and BA: writing—original draft. DB: conceptualization and writing—original draft. SD and LM: writing—final draft. All authors contributed to the article and approved the submitted version.

## References

[B1] Alikhademi K. Drobina E. Prioleau D. Richardson B. Purves D. Gilbert J. E.. (2021). A review of predictive policing from the perspective of fairness. Artif. Intell. Law 30, 1–17. 10.1007/s10506-021-09286-4

[B2] Asaro P. M. (2019). AI ethics in predictive policing: from models of threat to ethics of care. IEEE Technol. Soc. Mag. 38, 40–53. 10.1109/MTS.2019.2915154

[B3] Bettin B. Steelman K. Wallace C. Pontious D. Veinott E. (2022). Identifying and addressing risks in the early design of a sociotechnical system through premortem. Proc. 2022 HFES 66th Ann. Meet. 66, 1514–1518. 10.1177/1071181322661307

[B4] Blasch E. Sung J. Nguyen T. (2021). Multisource ai scorecard table for system evaluation. arXiv. [preprint] 2102.03985. 10.48550/arXiv.2102.03985

[B5] Borenstein J. Herkert J. R. Miller K. W. (2019). Self-driving cars and engineering ethics: the need for a system level analysis. Sci. Eng. Ethics 25, 383–398. 10.1007/s11948-017-0006-0 29134429

[B6] Cañas J. J. (2022). AI and ethics when human beings collaborate with AI agents. Front. Psychol. 13, 1–9. 10.3389/fpsyg.2022.836650 35310226 PMC8931455

[B7] Carayon P. Hancock P. Leveson N. Noy I. Sznelwar L. van Hootegem G.. (2015). Advancing a sociotechnical systems approach to workplace safety – developing the conceptual framework. Ergonomics 58, 548–564. 10.1080/00140139.2015.1015623 25831959 PMC4647652

[B8] Coeckelbergh M. (2020). AI Ethics. Cambridge, MA: MIT Press. 10.7551/mitpress/12549.001.0001

[B9] Cofone I. N. (2018). Servers and waiters: what matters in the law of A.I. Stanf. Technol. Law Rev. 21, 167–197. 10.31228/osf.io/2nstf

[B10] de Rosa F. De Gloria A. (2021). Design methodology of analytical games for knowledge acquisition. Int. J. Serious Games 8, 3–23. 10.17083/ijsg.v8i4.456

[B11] Degeling M. Berendt B. (2018). What is wrong about Robocops as consultants? A technology-centric critique of predictive policing. AI Soc. 33, 347–356. 10.1007/s00146-017-0730-7

[B12] Dorton S. L. (2022). Supradyadic trust in artificial intelligence. Artif. Intell. Soc. Comput. 28, 92–100. 10.54941/ahfe1001451

[B13] Dorton S. L. Harper S. B. Neville K. J. (2022). Adaptations to trust incidents with artificial intelligence. Proc. HFES 66th Int. Ann. Meet. 66, 95–99. 10.1177/1071181322661146

[B14] Dorton S. L. Harper S. H. (2022). A naturalistic investigation of trust, AI, and intelligence work. J. Cogn. Eng. Decis. Mak. 16, 222–236. 10.1177/15553434221103718

[B15] Dorton S. L. Maryeski L. R. Ogren L. Dykens I. T. Main A. (2020). A wargame-augmented knowledge elicitation method for the agile development of novel systems. Systems 8, 1–15. 10.3390/SYSTEMS8030027

[B16] Eckert T. (2015). “The pre-mortem: an alternative method of predicting failure,” in 2015 IEEE Symposium on Product Compliance Engineering (ISPCE) (Chicago, IL: IEEE), 1–4. 10.1109/ISPCE.2015.7138700

[B17] Ezer N. Bruni S. Cai Y. Hepenstal S. J. Miller C. A. Schmorrow D. D.. (2019). Trust engineering for human-AI teams. Proc. Hum. Factors Ergon. Soc. Ann. Meet. 63, 322–326. 10.1177/1071181319631264

[B18] Gallop D. Willy C. Bischoff J. (2016). How to catch a black swan: measuring the benefits of the premortem technique for risk identification. J. Enterp. Transform. 6, 87–106. 10.1080/19488289.2016.1240118

[B19] Hacker P. (2022). The European AI liability directives: critique of a heal-hearted approach and lessons for the future. arXiv. [preprint]. 2211.13960. 10.48550/arXiv.2211.13960

[B20] Hacker P. Krestel R. Grundmann S. Naumann F. (2020). Explainable AI under contract and tort law: legal incentives and technical challenges. Artif. Intell. Law 28, 415–439. 10.1007/s10506-020-09260-6

[B21] Hallamaa J. Kolliokoski T. (2022). AI ethics as applied ethics. Front. Comput. Sci. 4, 1–12. 10.3389/fcomp.2022.776837

[B22] Heimo O. I. Holvitie J. (2020). “Ethical debt in IS development, comparing ethical and technical debt,” in ETHICOMP 2020: Paradigm Shifts in ICT Ethics, eds J. Pelegrín-Borondo, M. Arias-Oliva, K. Murata, and M. L. Palma (Logroño, ES: Universidad de La Rioja), 29–31. Available online at: https://vbn.aau.dk/ws/files/413110611/Open_Access_Abstract.pdf#page=31

[B23] Hine E. Floridi L. (2023). The blueprint for an AI bill of rights: In search of enaction, at risk of inaction. Minds Mach. 10.1007/s11023-023-09625-1

[B24] Hoffman R. R. Ward P. Feltovich P. J. DiBello L. Fiore S. M. Andrews D. H.. (2014). Accelerated Expertise: Training for High Proficiency in a Complex World. London: Psychology Press. 10.4324/9780203797327

[B25] Husovec M. Laguna I. R. (2022). Digital services act: a short primer. SSRN. 10.2139/ssrn.4153796

[B26] Jefferies C. Balkin E. A. Groom L. Rayo M. (2022). Developing systemic contributors and adaptations diagramming (SCAD): systemic insights, multiple pragmatic implications. Proc. 2022 HFES 66th Int. Ann. Meet. 66, 75–79. 10.1177/1071181322661334

[B27] Klein G. (2007). Performing a project premortem. Harv. Bus. Rev. 85, 18–19.

[B28] Klein G. (2022). Snapshots of the Mind. Cambridge, MA: MIT Press. 10.7551/mitpress/14342.001.0001

[B29] Klein G. Ross K. G. Moon B. M. Klein D. E. Hoffman R. R. Hollnagel E.. (2003). Macrocognition. IEEE Intell. Syst. 18, 81–85. 10.1109/MIS.2003.1200735

[B30] Klein G. Wright C. (2016). Macrocognition: from theory to toolbox. Front. Psychol. 7, 1–5. 10.3389/fpsyg.2016.00054 26858684 PMC4731510

[B31] Klein G. A. Orasanu J. Calderwood R. Zsambok C. E. (1993). Decision Making in Action: Models and Methods. New York, NY: Ablex.

[B32] Miller K. W. (2011). Moral responsibility for computing artifacts: “The Rules”. IT Prof. 13, 57–59. 10.1109/MITP.2011.4629134429

[B33] Miró-Llinares F. (2020). Predictive policing: utopia or dystopia? On attitudes towards the use of big data algorithms for law enforcement. Rev. D'Internet Derecho Polit. 30, 1–18. 10.7238/idp.v0i30.3223

[B34] Mittelstadt B. D. Allo P. Taddeo M. Wachter S. Floridi L. (2016). The ethics of algorithms: mapping the debate. Big Data Soc. 3, 1–21. 10.1177/2053951716679679

[B35] Morey D. A. Rayo M. F. Li M. (2022). From reactive to proactive safety: joint activity monitoring for infection prevention. Proc. 2022 Int. Symp. Hum. Factors Ergon. Healthc. 11, 48–52. 10.1177/2327857922111009 36726370 PMC9888306

[B36] Munn L. (2022). The uselessness of AI ethics. AI Ethics. 10.1007/s43681-022-00209-w

[B37] Naturalistic Decision Making Association (NDMA) (n.d.). Principles of Naturalistic Decision Making. Available online at: https://naturalisticdecisionmaking.org/ndm-principles/ (accessed May 23 2023).

[B38] Nemeth C. Klein G. (2010). “The naturalistic decision making perspective,” in Wiley Encyclopedia of Operations Research and Management Science. SemanticScholar. 10.1002/9780470400531.eorms0410

[B39] Neville K. Pires B. Madhavan P. Booth M. Rosfjord K. Patterson E.. (2022). The TRUSTS work system resilience framework: a foundation for resilience-aware development and transition. Proc. 2022 HFES 66th Int. Ann. Meet. 66, 2067–2071. 10.1177/1071181322661177

[B40] Petrozzino C. (2021). Who pays for ethical debt in AI? AI Ethics 1, 205–208. 10.1007/s43681-020-00030-3

[B41] Richardson R. Schultz J. M. Crawford K. (2019). Dirty data, bad predictions: how civil rights violations impact police data, predictive policing systems, and justice. N. Y. Univ. Law Rev. Online 94, 15–55.

[B42] Selbst A. D. (2020). Negligence and AI's human users. Boston Univ. Law Rev. 100, 1315–1376.

[B43] Sheridan T. B. (2008). Risk, human error, and system resilience: fundamental ideas. Hum. Factors 50, 418–426. 10.1518/001872008X250773 18689048

[B44] Stein A. L. (2022). Assuming the risks of artificial intelligence. Boston Univ. Law Rev. 102, 979–1035.

[B45] Sullivan H. R. Schweikart S. J. (2019). Are current tort liability doctrines adequate for addressing injury caused by AI? AMA J. Ethics 21, 160–166. 10.1001/amajethics.2019.160 30794126

[B46] Veinott E. Klein G. A. Wiggins S. (2010). “Evaluating the effectiveness of the PreMortem technique on plan confidence,” in Proceedings of the 7th International ISCRAM Conference, Seattle, USA, May 2010.

[B47] Vogel K. M. Reid G. Kampe C. Jones P. (2021). The impact of AI on intelligence analysis: tackling issues of collaboration, algorithmic transparency, accountability, and management. Intell. National Secur. 36, 827–848. 10.1080/02684527.2021.1946952

[B48] Ward P. Gore J. Hutton R. Conway G. Robert H. (2018). Adaptive skill as the conditio sine qua non of expertise. J. Appl. Res. Mem. Cogn. 7, 35–50. 10.1016/j.jarmac.2018.01.009

[B49] Woods D. Dekker S. (2010). Anticipating the effects of technological change: a new era of dynamics for human factors. Theor. Issues Ergon. Sci. 1, 272–282. 10.1080/14639220110037452

[B50] Woods D. D. (2006). “The law of stretched systems in action: exploiting robots,” in Proceedings of the 1^st^ ACM SIGCHI/SIGART Conference on Human-Robot Interaction (Salt Lake City, UT), 1. 10.1145/1121241.1121242

